# Microfluidic Sorting Can Be Applied for Assisted Reproduction Sperm Selection in Different Cases of Semen Abnormalities

**DOI:** 10.3390/life15050790

**Published:** 2025-05-15

**Authors:** Giulia Traini, Maria Emanuela Ragosta, Lara Tamburrino, Alice Papini, Sarah Cipriani, Linda Vignozzi, Elisabetta Baldi, Sara Marchiani

**Affiliations:** 1 Department of Experimental and Clinical Biomedical Sciences “Mario Serio”, University of Florence, 50134 Florence, Italy; giulia.traini@unifi.it (G.T.); mariaemanuela.ragosta@unifi.it (M.E.R.); alice.papini@edu.unifi.it (A.P.); sarah.cipriani@unifi.it (S.C.); linda.vignozzi@unifi.it (L.V.); 2 Andrology, Women’s Endocrinology and Gender Incongruence Unit, Center for Prevention, Diagnosis and Treatment of Infertility, Careggi University Hospital, 50134 Florence, Italy; lara.tamburrino@unifi.it; 3 Aware Fertility Unit, AUSL Toscana Centro, 50122 Florence, Italy; 4 Department of Experimental and Clinical Medicine, University of Florence, 50134 Florence, Italy; elisabetta.baldi@unifi.it

**Keywords:** microfluidic selection, Swim-up techniques, sperm DNA fragmentation, TUNEL/PI assay, sperm oxidative levels, CellROX^®^ Orange probe, sperm chromatin compaction

## Abstract

Sperm preparation is a critical step in assisted reproduction, aiming to isolate spermatozoa with optimal characteristics and high fertilizing potential. Traditional sperm selection methods involve centrifugation, which may cause sperm damage. Microfluidic sperm sorting (MSS) offers an alternative approach, mimicking the female reproductive tract environment, avoiding centrifugation, and reducing manipulation and processing time. This study aims to compare the performance of MSS and Swim-up (SU) in 26 normozoospermic, 31 hyperviscous normozoospermic, 15 oligozoospermic, and 9 asthenozoospermic subjects. Semen samples were collected from male subjects undergoing routine semen analysis at Careggi University Hospital, Florence. Sperm selection was carried out using both SU and MSS. The parameters assessed included sperm motility, viability, concentration, kinematics, DNA fragmentation (sDF), chromatin compaction, and oxidative status. Both SU and MSS improved sperm characteristics compared to unselected samples. MSS isolated high-quality spermatozoa with lower sDF and higher chromatin compaction than SU, not only in normozoospermic samples but also in samples with semen defects like hyperviscosity, low concentration and/or motility, and high sDF. In conclusion, the use of microfluidics may enhance the chances of successful fertilization and improve reproductive outcomes, especially for individuals with compromised semen quality where conventional methods may fail.

## 1. Introduction

The use of Assisted Reproductive Technology (ART) represents an effective treatment option, offering infertile couples the opportunity to build a family. Sperm preparation is a crucial step in ART procedures, as it aims to isolate spermatozoa with optimal characteristics and high fertilizing potential, ultimately contributing to a successful pregnancy [1]. Common in vitro sperm preparation techniques bypass the natural selection barriers that spermatozoa encounter as they pass through the female reproductive tract. These methods primarily select spermatozoa based on their motility and morphology, while neglecting other important mechanisms, such as rheotaxis, thermotaxis, and chemotaxis, which contribute to sperm selection in vivo [2,3].

Currently, in ART laboratories, two sperm selection methods, Density Gradient Centrifugation (DGC) and Swim-up, are routinely used. These methods are based, respectively, on sedimentation or migration to separate spermatozoa [4]. Swim-up selects spermatozoa based on their ability to swim from the seminal plasma to the culture medium. This method may be performed by layering the culture medium directly over the semen (direct Swim-up) or layering the culture medium over the pellet, which is obtained after the centrifugation of the sample (pellet Swim-up). DGC separates sperm cells based on their density by centrifuging semen over a density gradient [5]. Both techniques isolate sperm cells from other semen constituents, such as germ cells, leucocytes, epithelial cells, unviable spermatozoa, or debris. The choice of selection method, according to the WHO manual [5], depends on semen quality. Swim-up is usually preferred for subjects with normal seminal parameters, whereas DGC is commonly used for cases of oligo-, terato-, or asthenozoospermia, as it recovers a higher number of motile spermatozoa [5].

Centrifugation steps in both DGC and pellet Swim-up may lead to an increase in reactive oxygen species (ROS), resulting in plasma membrane peroxidation and, ultimately, sperm DNA damage [6,7]. Several studies have reported elevated levels of sperm oxidative stress and DNA fragmentation after selection, particularly following DGC [6,8,9,10,11,12].

Other drawbacks of both methods include reduced yields in abnormal semen samples and a long processing time. Alternative separation techniques have been developed based on the surface and binding properties of spermatozoa; however, their use in clinical practice is currently limited, as they share the same disadvantages [13] and are quite cumbersome.

In recent years, microfluidics has begun to be used to isolate gametes, embryos, and other cell types. This method takes advantage of miniaturization by using microchannels with dimensions of a few hundred micrometers, enabling the manipulation of small quantities of fluids. In case of sperm separation, this method appears highly promising since (1) it mimics the natural in vivo environment of the female reproductive tract; (2) it preserves spermatozoa against damage by excluding centrifugation and reducing the processing time; and (3) it can be designed to combine different separation methods within the same device [14].

In recent years, different microfluidic devices have been developed inspired by parallel laminar flow stream to distinguish motile from non-motile spermatozoa and other cellular debris [15,16,17], via sperm rheotaxis, which allows spermatozoa to swim against the flow [7,18,19,20,21], and via sperm responsiveness to a chemotactic gradient [22,23,24,25]. Other microfluidic devices are based on sperm responsiveness to temperature gradient [26,27] or on a combination of chemotactic and temperature stimuli [3,28,29]. However, to date, major drawbacks have hindered the introduction of most of these systems into the consumer market.

Currently, only three microfluidic chips are commercially available. All are made of polymers (polymethyl-methacrylate or polycarbonate) and consist of a single inlet channel, in which the sample is placed, communicating with a lower chamber. A microporous membrane filter separates the lower chamber from the upper chamber, which is filled only with medium. Only highly motile and morphologically normal spermatozoa can reach the upper outlet chamber, where they are collected. Published studies to date agree that sperm quality improves after microfluidic sorting compared to both raw semen and other selection methods (DGC or Swim-up) [30,31,32]. However, most of these studies were conducted almost exclusively on normozoospermic subjects, where conventional sperm selection techniques can also yield good results. The effectiveness of microfluidics for categories of subjects with abnormal semen parameters has been scarcely investigated so far [32,33,34].

While microfluidics has been applied in ART in some studies, it remains unclear whether sperm selection through microfluidic sperm sorting (MSS) leads to significantly improved ART outcomes. A recent meta-analysis suggested a modest improvement in clinical parameters, but the results remained inconclusive [35] due to the heterogeneity of included studies concerning inclusion/exclusion criteria and experimental design. Other studies [36,37,38,39] have supported the potential benefits of MSS, particularly in increasing the number of euploid embryos in couples who failed a previous ART cycle.

The aim of this study was to compare MSS with the conventional pellet Swim-up (SU) technique by evaluating standard semen parameters, sperm kinematic parameters, DNA fragmentation (sDF), and oxidative status in normozoospermic (*n* = 26), oligozoospermic (*n* = 15), and asthenozoospermic (*n* = 9) samples. Additionally, the two techniques were compared in hyperviscous normozoospermic semen samples (*n* = 31), for which conventional separation techniques often fail to obtain good yields. The comparison between the two methods was carried out by using exactly the recommended standard procedures for each of the two techniques.

## 2. Materials and Methods

### 2.1. Chemicals

The flushing medium was purchased from CooperSurgical (Origio Italia Srl, Rome, Italy). PBS was obtained from Biosigma S.p.A. (Venice, Italy). The CellROX^®^ Orange Reagent, Yo-Pro-1 (Y1), and Propidium Iodide (PI) were purchased from Invitrogen by Thermo Fisher Scientific (Waltham, MA, USA). The In Situ Cell Death Detection Kit was purchased from Roche Molecular Biochemicals (Milan, Italy). Chromomycin A3 (CMA3) was obtained from Merck Life Sciences S.r.l. (Milan, Italy).

### 2.2. Human Semen Samples

This study was approved by the local ethical committee (Ref: 23266_bio). After the subjects signed informed consent, a total of 77 semen samples were included in the study, consisting of 26 normozoospermic, 31 hyperviscous normozoospermic, 15 oligozoospermic, and 9 asthenozoospermic samples. Four subjects were classified as both oligozoospermic and asthenozoospermic (oligoasthenozoospermic). Sample viscosity was assessed according to the WHO manual [5] by aspirating the semen into a pipette and allowing it to drop by gravity. If the sample formed a thread longer than 2 cm, it was classified as hyperviscous.

Semen samples were collected after an abstinence period of 2–7 days by masturbation from male subjects undergoing routine semen analysis for couple infertility at the Andrology Laboratory of Careggi University Hospital of Florence. Semen analysis was performed 30–60 min after collection according to the World Health Organization manual [5].

Sperm concentration was assessed using an improved Neubauer chamber after appropriate dilution of the sample in formalin-containing buffer using an optical microscope (Nikon Eclipse Ci, Nikon Europe B.V., Amstelveen, The Netherlands). Sperm motility was assessed by observing at least 200 spermatozoa per sample and classifying them as rapid progressive, slow progressive, non-progressive, and immotile spermatozoa. The percentages of rapid and slow progressive motile spermatozoa were added to obtain the percentage of total progressive motile spermatozoa. Sperm motility was evaluated by an optical microscope with a 37 °C heated plate, using a 40× objective. The eosin test was used to evaluate sperm viability by distinguishing between viable cells (with white-stained heads) and non-viable cells (with pink-stained heads). Viability was assessed by observing at least 200 spermatozoa per sample using an optical microscope. Sperm morphology was assessed after Diff-Quik staining, determining the percentage of normal and abnormal forms by scoring at least 100 spermatozoa per slide using an optical microscope with a 100× magnification objective.

Manual sperm analysis was performed by highly trained operators who have participated in the UK-NEQAS (United Kingdom National External Quality Assessment Service) external quality control program for semen analysis since 2005. The mean (±SD) percent biases for the laboratory for the year 2024 were 3.2 (±12.7) for progressive motility, 4.7 (±11.8) for total motility, and 3.4 (±6.9) for sperm concentration (*n* = 16, data from UK-NEQAS).

For each sample, all analyses were performed on raw semen (unselected) and spermatozoa selected using both SU and MSS.

### 2.3. Microfluidic Sperm Sorting

MSS was performed using the ZyMōt Multi 850 µL Sperm Separation Device (CooperSurgical, Origio Italia Srl, Rome, Italy). Briefly, 850 μL of the semen sample was loaded into the inlet port of the device by using a syringe. Then, 750 µL of the Flushing medium was placed on the upper chamber to cover the entire membrane surface. The loaded device was incubated at 37 °C for 30 min, and then spermatozoa were retrieved from the outlet port by aspirating 500 µL.

### 2.4. Pellet Swim-Up Technique

In total, 850 μL of the semen sample was centrifuged at 500× *g* for 10 min in an equal volume of the Flushing medium. The supernatant was then removed, and 1 mL of fresh Flushing medium was gently layered over it. The sample was incubated for 1 h at 37 °C. After incubation, only the upper fraction (approximately 500 µL) containing motile spermatozoa was carefully collected into a new tube.

In five semen samples, the SU technique was performed using incubation periods of 30 and 60 min. Following incubation, the upper fraction was collected, and sperm motility, viability, concentration, and DNA fragmentation were assessed.

### 2.5. Direct Swim-Up Technique

Direct Swim-up selection was performed by layering 1 mL of the Flushing medium over 850 μL of whole semen and incubating at 37 °C for 1 h. Then, the upper fraction (approximately 500 µL) containing the motile fraction of spermatozoa was collected into a new tube.

### 2.6. Assessment of Sperm Intracellular ROS

Intracellular ROS were detected using the CellROX^®^ Orange probe, as previously described [40]. Briefly, 4 × 10^6^ spermatozoa were washed in the Flushing medium by centrifugation at 500× *g* for 5 min and divided into two equal aliquots. One aliquot was incubated in 200 μL of PBS with 1 μM CellROX^®^ Orange added, while the other aliquot was incubated with the medium only, both for 30 min at 37 °C and 5% CO_2_. After incubation, the samples were washed three times with PBS, resuspended in 300 μL of PBS, and Y1 (2.5 nM) was added for acquisition by flow cytometry.

### 2.7. Assessment of Sperm DNA Fragmentation

sDF was detected by TUNEL (terminal deoxynucleotidyl transferase (TdT)-mediated FITC-dUTP nick end labeling) assay using the In Situ Cell Death Detection Kit (Roche Molecular Biochemicals, Milan, Italy) with the protocol published by Muratori et al. [41], but with slight modifications. First, 3 × 10^6^ spermatozoa were fixed in 150 μL of 4% paraformaldehyde (in phosphate-buffered saline (PBS) pH 7.4), centrifuged at 500× *g* for 5 min, washed twice with 200 μL of PBS with 1% BSA, and permeabilized in 30 μL of 0.1% sodium citrate buffer with 0.1% Triton X-100 of for 4 min in ice. Then, the samples were divided into two aliquots for the labeling reaction. The test sample was incubated in the labeling solution (supplied with the kit) with the TdT enzyme (diluted 1:10) for 1 h at 37 °C in the dark. The negative control was prepared by omitting TdT. Finally, samples were washed twice, resuspended in 300 μL of PBS, stained with PI (50 µg/mL), and analyzed with a flow cytometer.

### 2.8. Flow Cytometry

Samples were acquired with a FACScan flow cytometer (BD Biosciences, San Jose, CA, USA) equipped with a 15-mW argon-ion laser for excitation. A total of 8000 events in the characteristic forward scatter/side scatter region of spermatozoa [41] were acquired. Green fluorescence of TUNEL and Y1 and red fluorescence of CellROX^®^ Orange and PI were revealed with the FL-1 (515–555 nm wavelength band) and FL-2 (563–607 nm wavelength band) detectors, respectively. A marker including 99% of the total events was established in the dot plot of the negative controls and translated to the corresponding test sample. All the events beyond the marker were considered positive. Data analysis was performed with the CellQuest-Pro software program version 5.2.1 (BD Biosciences, Franklin Lakes, NJ, USA). The percentage of positive spermatozoa to CellROX^®^ Orange was determined within the Y1-negative events of the characteristic forward-scatter/side-scatter region of spermatozoa (viable spermatozoa, [40]). The percentage of DNA-fragmented spermatozoa was determined within PI-positive events of the characteristic forward-scatter/side-scatter region of spermatozoa. The percentage of TUNEL-positive spermatozoa was calculated within the PI brighter population (containing both viable and unviable spermatozoa, as well as both DNA-fragmented and non-DNA-fragmented spermatozoa), the PI dimmer population (containing unviable and DNA-fragmented spermatozoa), and in both sperm populations (total sDF) [41].

### 2.9. Assessment of Sperm Kinematic Parameters and Hyperactivated Motility

Raw or selected samples were analyzed using Computer-Assisted Sperm Analysis (C.A.S.A., Hamilton Thorne Research CEROS II, Beverly, MA, USA). The following kinematic parameters were recorded: average path velocity (VAP, μm/s), straight line velocity (VSL, μm/s), curvilinear velocity (VCL, μm/s), amplitude of lateral head displacement (ALH, μm), beat cross frequency (BCF, Hz), straightness (STR, %), and linearity of progression (LIN, %). The settings used for evaluation were an analysis duration of 1s (30 frames); maximum and minimum head sizes of 50 and 5 μm^2^; minimum head brightness of 170; and minimum tail brightness of 70. A minimum of 200 motile cells and 5 fields were analyzed for each aliquot. According to Mortimer et al. [42], the threshold values of VCL ≥ 150 μm/s, ALH ≥ 7 μm, and LIN ≤ 50% were set to identify a fraction representing the percentage of hyperactivated spermatozoa (HA, %).

### 2.10. Assessment of Sperm Chromatin Compaction by Chromomycin A3

Sperm chromatin compaction was evaluated with CMA3, a fluorochrome that competes with protamines for binding to the minor groove of GC-rich DNA [43], but only in oligozoospermic samples because the recovery after selection was not enough to evaluate sperm DNA fragmentation via TUNEL/PI assay.

First, 0.4 × 10^6^ spermatozoa were fixed in 4% paraformaldehyde (in phosphate-buffered saline (PBS), pH 7.4) for 30 min at room temperature. Afterward, they were incubated with 100 μL of the CMA3 solution (0.25 mg/mL in McIlvane’s buffer (0.2 M Na_2_HPO_4_, 0.1 M citric acid), pH 7.0, containing 10 mM MgCl_2_) for 20 min at room temperature in the dark. After washing, spermatozoa were resuspended in 10 μL of McIlvane’s buffer, pH 7.0, containing 10 mM MgCl_2_, smeared on a slide, air-dried, and mounted with PBS–glycerol (1:1). Two hundred spermatozoa were analyzed on each slide using a fluorescence microscope (Axiolab A1 FL; Carl Zeiss, Milan, Italy) with an oil immersion 100× magnification objective [39]. Two types of staining patterns were identified: bright green sperm heads (indicating low protamine content and abnormal chromatin packaging) and weak green sperm heads (indicating high protamine content and normal chromatin packaging) [44].

### 2.11. Statistical Analysis:

A post hoc sample size calculation revealed that the sample size used for each group was sufficient to assess sperm sorting efficiency between MSS and SU with α = 0.05 and above 90% power. Since data followed a non-normal distribution, as verified by the Kolmogorov–Smirnov test, they were expressed as median values (interquartiles, IQR). The Wilcoxon signed-rank test was used for comparison among groups. A *p*-value of 0.05 was considered significant. All statistical analyses were performed using the Statistical Package for the Social Sciences version 29.0 (SPSS, Chicago, IL, USA) for Windows.

## 3. Results

### 3.1. Comparison of MSS and SU Selection Methods in Hyperviscous and Normally Viscous Semen Samples

First, to assess the yield after microfluidic selection of highly viscous semen samples, we divided the 57 normozoospermic samples into two groups based on viscosity and performed statistical analysis separately for each group. The age, sexual abstinence, and standard semen parameters of the normozoospermic samples with normal viscosity (*n* = 26) and hyperviscosity (*n* = 31) are reported in Table 1. No statistically significant differences in the baseline parameters were observed between samples with normal viscosity and those with hyperviscosity (Table 1).

**Figure 1:**
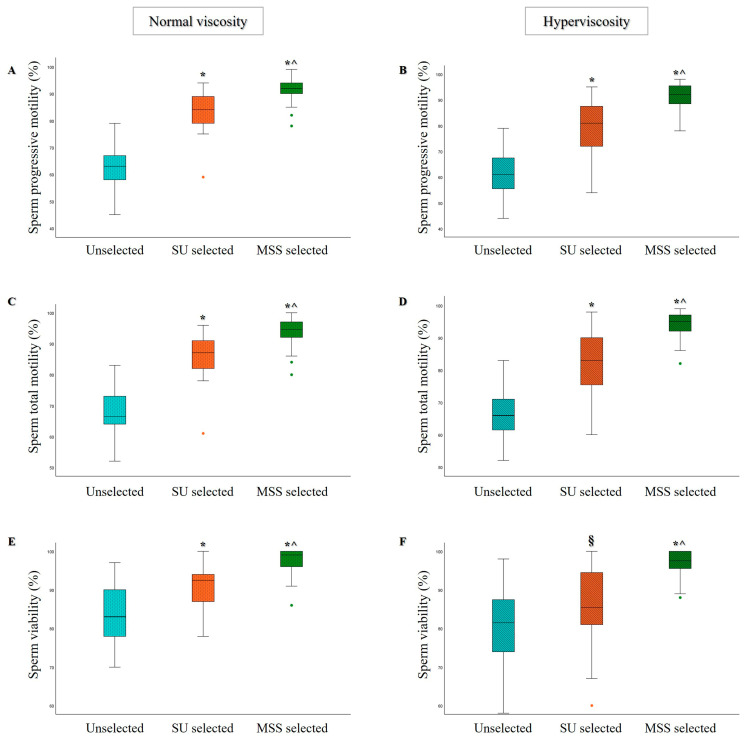
Box plots representing the median values of sperm progressive motility (**A**,**B**), total motility (**C**,**D**), and viability (**E**,**F**) in Unselected, SU, and MSS selected samples, for the normal viscosity (*n* = 26) and hyperviscosity (*n* = 31) groups. § *p* < 0.05 vs. Unselected; * *p* < 0.001 vs. Unselected; ^ *p* < 0.001 vs. SU selected.

As expected, compared to unselected samples, a significant increase in the percentage of sperm progressive motility (Figure 1A,B), total motility (Figure 1C,D), and viability (Figure 1E,F) was observed after selection with both MSS and SU techniques in both groups of normozoospermic samples. Notably, a statistically significant improvement in all parameters was observed after MSS selection compared to SU (Figure 1) in both non-viscous and hyperviscous semen samples.

**Figure 2:**
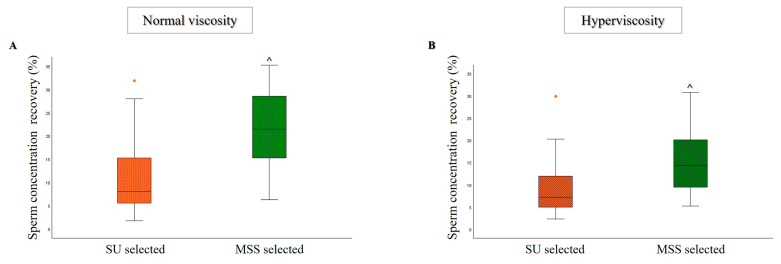
Box plots representing sperm concentration recovery rate (calculated as yield relative to unselected samples) after SU and MSS selection, for the normal viscosity ((**A**), *n* = 26) and hyperviscosity ((**B**), *n* = 31) groups. ^ *p* < 0.001 vs. SU selected.

Although both selection procedures were performed using the same starting volume of semen samples, a statistically significant increase in sperm concentration was observed with MSS compared to the SU technique in both groups of semen samples (Figure 2A,B).

**Figure 3:**
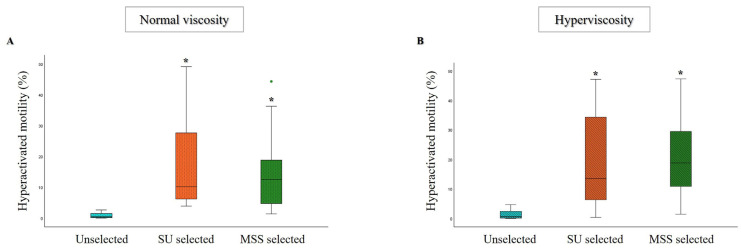
Box plots representing the median values of hyperactivated motility in unselected, SU, and MSS selected samples, for the normal viscosity ((**A**), *n* = 26) and hyperviscosity ((**B**), *n* = 31) groups. * *p* < 0.001 vs. Unselected.

An increase in the percentage of hyperactivated spermatozoa was observed after MSS and SU selection compared to unselected samples, both in normally viscous and hyperviscous samples. The percentage of hyperactivated spermatozoa did not differ significantly between the two selection methods, although a slight increase in average levels was noted after MSS (Figure 3A,B). Additionally, kinematic parameters (such as VAP, VSL, VCL, ALH, BCF, LIN, and STR) showed improvement after selection with both techniques (Table 2). Interestingly, a notable enhancement in most of the kinematic parameters following MSS compared to SU was observed in both groups of semen samples (Table 2).

**Figure 4:**
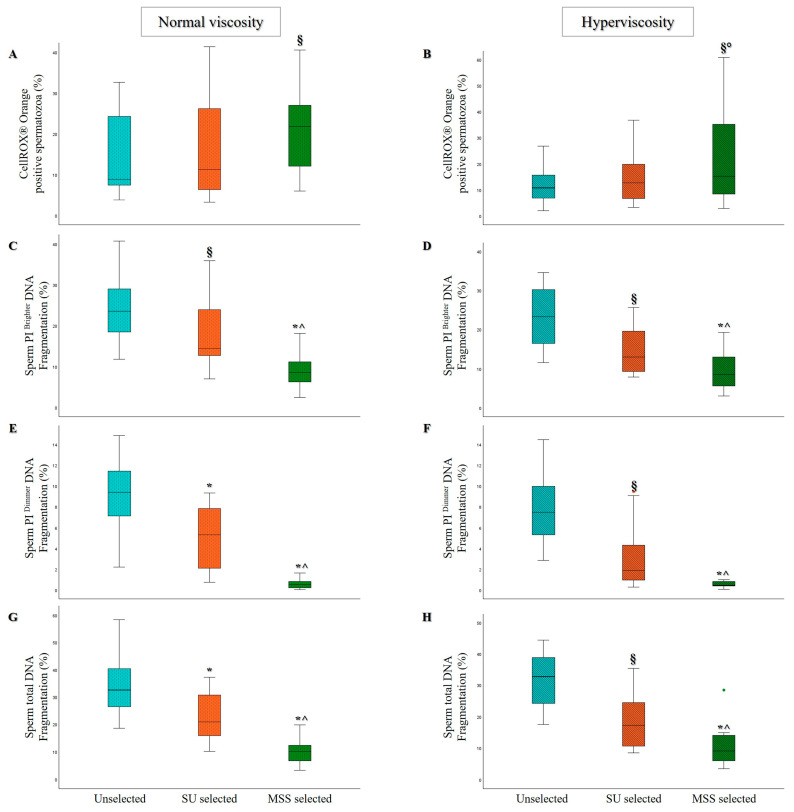
Box plots representing the median values of CellROX^®^ Orange sperm positivity (which is able to detect oxidized sperm fraction related to better parameters [40]; (**A**,**B**)), PI brighter (**C**,**D**), PI dimmer (**E**,**F**), and total (**G**,**H**) sDF in Unselected, SU, and MSS selected samples, for the normal viscosity (*n* = 11 for sperm oxidation and *n* = 19 for sDF) and hyperviscosity (*n* = 12 for sperm oxidation and *n* = 15 for sDF) groups. § *p* < 0.05 vs. Unselected; * *p* < 0.001 vs. Unselected; ° *p* < 0.05 vs. SU selected; ^ *p* < 0.001 vs. SU selected.

In 23 semen samples (11 with normal viscosity and 12 with hyperviscosity), we evaluated oxidative levels using the CellROX^®^ Orange probe, which was recently shown by our group to identify the sperm viable fraction with better characteristics [40]. A statistically significant increase in the percentage of oxidized spermatozoa was observed in MSS samples compared to unselected samples (Figure 4A,B) in both non-viscous and hyperviscous semen samples. Notably, in hyperviscous samples, CellROX^®^ Orange sperm positivity significantly increased after MSS selection, compared to both unselected and SU-selected samples (Figure 4B).

Both oxidation levels (Figure 4A,B) and sDF (Figure 4C–H) improved significantly after MSS.

Lastly, we evaluated sDF in 34 semen samples (19 with normal viscosity and 15 with hyperviscosity). As shown in Figure 4C–H, a statistically significant decrease in sDF was observed in the PI brighter, PI dimmer, and total sperm population with both selection methods compared to the unselected samples, although MSS yielded significantly lower sDF levels compared to SU in both normally viscous and hyperviscous samples.

**Figure 5:**
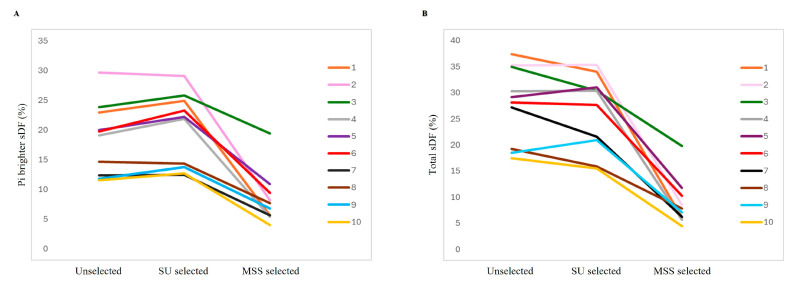
Line graphs showing individual values of PI brighter (**A**) and total (**B**) sDF in 10 out 34 semen samples in which SU selection did not improve sDF whereas MSS reduced it.

Noteworthy, MSS is able to decrease both PI brighter and total sDF, even in samples with sDF levels above the cutoff values discriminating fertile and sub-fertile men with our method (PI brighter sDF = 17.5%, total sDF = 30.5%, [45]). This reduction was similar to that observed in semen samples with sDF below cutoff values (PI brighter sDF: −63% for samples above cutoff vs. −54% for samples below cutoff; total sDF: −71% for samples above cutoff vs. −74% for samples below cutoff). Notably, in 10 out of 34 samples, SU selection did not improve or even increase sDF, whereas MSS reduced it in all samples (Figure 5). On average, the decrease was −52% for PI brighter and −64% for total sperm populations.

To verify that the improvement in the analyzed sperm parameters observed after MSS compared to SU is not due to the centrifugation step included in the SU indirect protocol, we compared MSS and SU with direct SU in 18 normozoospermic samples. As shown in Table 3, a statistically significant increase in standard semen parameters, particularly sperm progressive and total motility and viability, was observed in MSS selected samples compared to both direct SU and SU selected samples. MSS also improved some kinematic parameters (ALH, BCF, and VCL) compared to direct SU, whereas the percentage of hyperactivated spermatozoa was similar after selection with all three procedures. A slight, not statistically significant increase in sperm oxidation was observed in MSS selected samples compared to the other groups (Table 3). Conversely, a statistically significant decrease was observed in MSS samples compared to both direct and indirect SU selected samples for PI Brighter, PI Dimmer, and total sDF (Table 3).

Overall, only small, non-significant differences were observed between direct and indirect SU in post-selection sperm parameters, indicating that the ameliorative effect of MSS is not due to the centrifugation step.

### 3.2. Comparison of MSS and SU Selection Methods in Oligozoospermic and Asthenozoospermic Samples

The age, sexual abstinence, and semen parameters of oligo- and asthenozoospermic samples are reported in Table 4.

**Figure 6:**
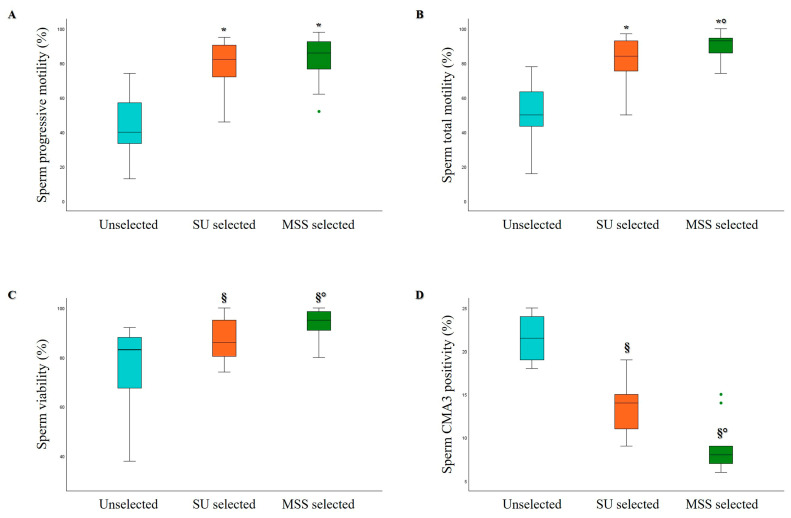
Box plots representing the median values of sperm progressive motility (*n* = 15, (**A**)), total motility (*n* = 15, (**B**)), viability (*n* = 15, (**C**)), and CMA3 positivity (*n* = 10, (**D**)) in Unselected, SU, and MSS selected oligozoospermic samples. § *p* < 0.05 vs. Unselected; * *p* < 0.001 vs. Unselected; ° *p*< 0.05 vs. SU selected.

As shown in Figure 6, in oligozoospermic samples (which, in our cohort, were characterized by a sperm concentration of less than 10 × 10^6^/mL), both selection methods significantly increased sperm progressive motility (Figure 6A), total motility (Figure 6B), and viability (Figure 6C) compared to unselected samples. However, MSS significantly increased the percentage of total motile and viable spermatozoa compared to SU, whereas no significant difference was observed in the percentage of progressive motile spermatozoa. Concerning sperm kinematic parameters, a significant improvement was found, particularly for VAP, VCL, VSL, ALH, BCF, and hyperactivated motility, with both selection methods compared to unselected samples. However, no differences were noted between MSS and SU.

Due to the low number of selected spermatozoa obtained from oligozoospermic samples, the evaluation of sperm oxidative status and sDF was not feasible. However, we assessed the degree of protamination using CMA3 staining, a test that can be performed with a low number of cells, to measure chromatin integrity and compaction [46]. A statistically significant decrease in sperm CMA3 positivity was observed after MSS selection compared to both unselected and SU selected samples (Figure 6D).

**Figure 7:**
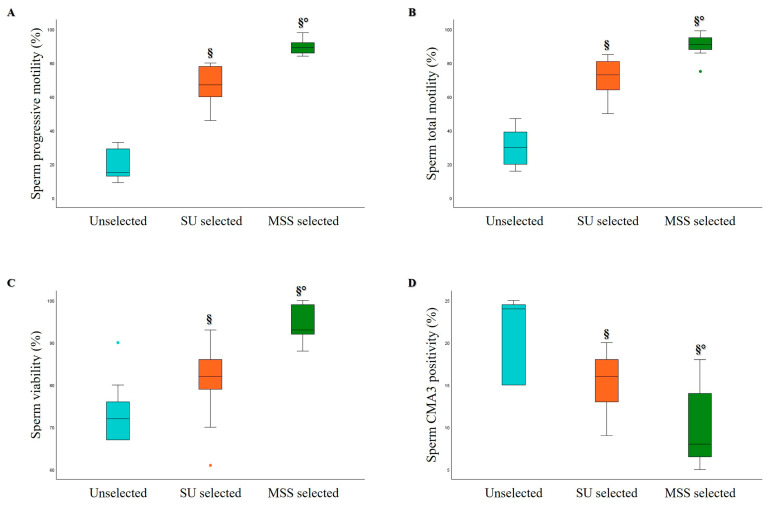
Box plots representing the median values of sperm progressive motility (*n* = 9, (**A**)), total motility (*n* = 9, (**B**)), viability (*n* = 9, (**C**)), and CMA3 positivity (*n* = 7, (**D**)) in Unselected, SU, and MSS selected asthenozoospermic samples. § *p* < 0.05 vs. Unselected; ° *p* < 0.05 vs. SU selected.

The comparison between MSS and SU was also conducted in 9 asthenozoospermic samples, characterized by progressive motility of less than 32% (Table 4). The data obtained for this category of samples were similar to those observed in normo- and oligozoospermic samples, showing a significant increase in sperm progressive motility (Figure 7A), total motility (Figure 7B), viability (Figure 7C), kinematic parameters, and hyperactivated motility after MSS selection compared to both unselected and SU selected samples.

Moreover, a significant decrease in sperm CMA3 positivity was observed in MSS selected samples compared to both unselected and SU samples (Figure 7D).

Of the 15 oligozoospermic and 9 asthenozoospermic samples, 4 belonged to both categories, characterized by both a low concentration and low motility. In this group, MSS was more effective in isolating spermatozoa with good motility, viability, and chromatin compaction compared to SU.

**Figure 8:**
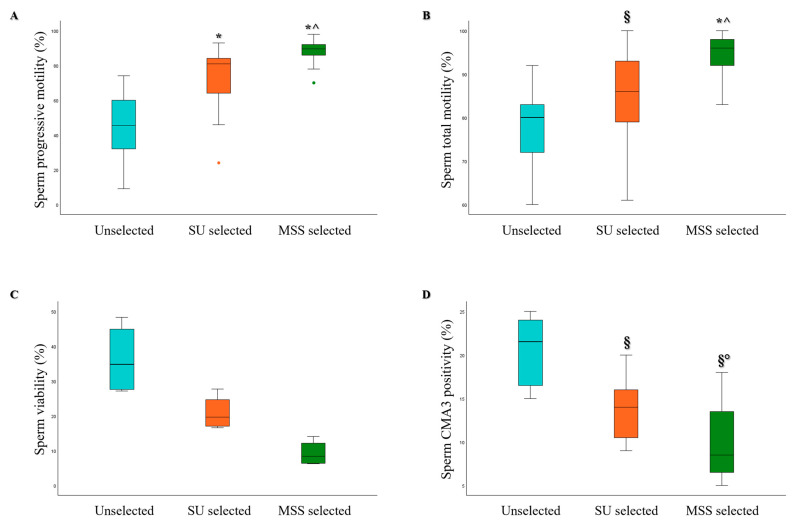
Box plots representing the median values of sperm progressive motility (**A**), viability (**B**), total sDF (**C**), and CMA3 positivity (**D**) in Unselected, SU, and MSS selected samples, for the teratozoospermic group (*n* = 26). § *p* < 0.05 vs. Unselected; * *p* < 0.001 vs. Unselected; ° *p* < 0.05 vs. SU selected; ^ *p* < 0.001 vs. SU selected.

Furthermore, when we extracted the subgroup of samples with normal morphology below 4% from our cohort, we identified 26 cases. Even within this subgroup, the MSS selection method led to significant improvements in sperm progressive motility, viability, DNA fragmentation, and CMA3 positivity compared to the SU method (Figure 8).

**Figure 9:**
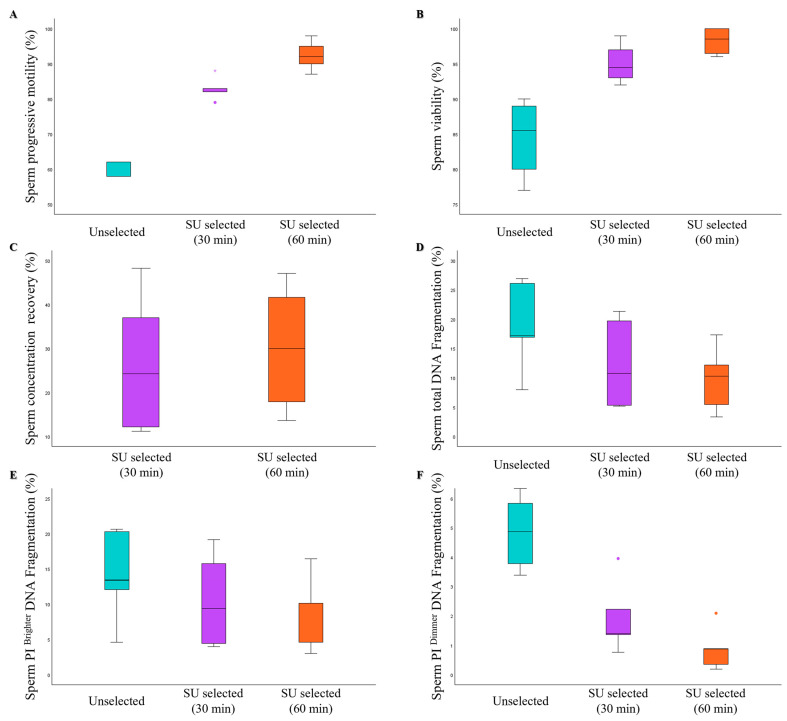
Box plots representing the median values of sperm progressive motility (**A**), viability (**B**), concentration recovery (**C**), total sDF (**D**), PI brighter sDF (**E**), and PI dimmer sDF (**F**) in Unselected, SU selected samples (30 min of incubation), and SU selected samples (60 min of incubation), *n* = 5; * *p* < 0.001.

To assess whether the extended incubation time during the SU procedure with respect to that of MSS may be responsible for the lower sperm quality found in the former, five samples were processed by SU with both 30- and 60-min incubation. No significant differences were observed in the percentages of progressive motility (Figure 9A), viability (Figure 9B), and DNA fragmentation (Figure 9D–F) of spermatozoa between the two time points. As expected, sperm concentration recovery was higher after 60 min of incubation compared to 30 min, although this difference did not reach statistical significance (Figure 9C).

## 4. Discussion

In recent years, the number of couples seeking ART procedures has increased, making the selection of high-quality spermatozoa crucial for improving fertilization outcomes [47]. Traditional sperm selection methods, such as Swim-up and DGC, can elevate ROS levels and cause DNA damage, including sDF [7,8,9,10]. Recently, attention has shifted to novel sperm selection techniques, particularly microfluidic systems, which do not require centrifugation. This approach aims to select live, motile, and morphologically normal spermatozoa, closely replicating the natural selection process in the female reproductive system.

In this study, we demonstrated that the microfluidic sorting of normo-, oligo-, terato-, and asthenozoospermic semen samples effectively identifies and selects a high-quality sperm fraction, characterized by elevated motility and viability, with virtually no immotile spermatozoa. This high quality of selected spermatozoa is evident not only when compared to the unselected samples but also in comparison to the conventional Swim-up method, both with (indirect) or without (direct) the centrifugation step. These findings are consistent with most studies in the literature [30,48,49,50], extending them to non-normozoospermic and high viscosity semen samples. Additionally, we evaluated the effect of MSS selection on kinetic parameters, corroborating results obtained for motility. To our knowledge, kinematic parameters after MSS selection were only reported in a previous study [32], with similar results as ours for VCL, LIN, and ALH. However, despite the improvement in kinematic parameters after MSS compared to SU, the percentage of hyperactivated spermatozoa did not statistically differ between the two selection methods, suggesting that the fraction of hyperactivated spermatozoa in semen samples is limited and that most, if not all, hyperactivated spermatozoa are selected with both methods. It is likely that the onset of hyperactivation is not due to the separation method but rather to the components present in the medium.

In our hands, the microfluidic method has been shown to yield higher or comparable sperm concentrations compared to the traditional approach. This result contrasts with those of other studies [31,32,33,50,51], likely due to differences in the initial semen volume used to compare the different methods. For example, in the study by Hsu et al. [32], the initial volume of seminal fluid used was 1 mL for DGC and 850 μL for microfluidics, resulting in a higher yield after traditional selection. Similarly, Pujoi et al. [31], Vahidi et al. [33], Zaha et al. [50], and Quinn et al. [51] observed significantly different sperm concentrations between MSS and other methods, likely because they used a microfluidic device where only 50 µL or less of seminal fluid was loaded. In contrast, Aydin et al. [52], starting from the same initial volume for both MSS and pellet Swim-up, found no differences in concentration recovery after selection. The fact that we recovered a higher sperm number, starting from the same semen volume, suggests that this procedure may be particularly useful for selecting healthy spermatozoa for IUI, where outcomes are highly dependent on the number of inseminated spermatozoa [53].

In line with previous studies [32,36,37,51], a distinct decrease in sDF was observed following selection, with a pronounced reduction using the microfluidic method. Our results demonstrated that MSS is capable of isolating spermatozoa with sDF levels below the cutoff values defined with our method [45], even when the sDF in neat semen was particularly high. Additionally, MSS selects spermatozoa with the correct chromatin structure, consistent with a recent study by Vahidi et al. [33]. High levels of sDF and an abnormal chromatin structure have been associated with male infertility, poor ART outcomes, and recurrent miscarriages [54,55,56,57,58,59,60,61].

As previously mentioned, studies [6,8,9,10,11,12] have shown that traditional sperm selection methods can increase sDF in some individuals while decreasing it in others ([62,63,64] and the present study), indicating that these procedures do not always select spermatozoa with minimized DNA damage. The centrifugation steps involved in both DGC and Swim-up procedures may increase ROS levels, which can explain the rise in DNA breakage after selection ([6,8,9,10,11,12] and the present study). It has also been shown that transition metals present in selection media may contribute to DNA damage [65]. In contrast, microfluidic sorting, which does not involve centrifugations and prior washing in preparation media and allows for rapid selection, is able to reduce sDF even in cases where traditional methods are less effective (present study, [32,51,66]). Here, we demonstrated that even in cases where the Swim-up procedure either increased or did not reduce sDF, MSS was able to improve this parameter (see Figure 5; [9,10]). Other alternative sperm selection strategies aimed at improving sperm quality prior to fertilization procedures involve the use of functionalized nanoparticles to selectively isolate spermatozoa with enhanced viability, DNA integrity, and membrane functionality. Evidence from animal studies suggests that such approaches may increase conception rates [67]. In human ART, compared to conventional methods such as DGC, nanoparticle-based selection has been associated with improved ICSI outcomes, including higher fertilization rates and an increased proportion of high-quality blastocysts [68].

Oxidative stress has a detrimental effect on semen parameters and fertility potential [69]. Several studies have reported higher levels of ROS in the semen of infertile men when compared to fertile controls [70]. However, it is well established that low levels of ROS are required for capacitation and the development of hyperactivated motility [71,72]. Here, we observed a significant increase in the percentage of CellROX^®^ Orange-positive spermatozoa after microfluidic selection compared to SU. Although this increase may appear contradictory with respect to several studies reporting an adverse effect of oxidative stress on sperm functions (for rev. see [73]), it likely reflects the selection of a functionally competent sperm subpopulation rather than indicative of cellular damage [40]. Indeed, as demonstrated by our group [40], this probe effectively detects oxidation levels reflecting better sperm performance in their reproductive functions, further suggesting that MSS selects spermatozoa with better performance and quality.

Only one study [30] has investigated the effect of selection through MSS on oxidative stress in human spermatozoa by comparing the oxidative reduction potential (ORP) across three different sperm selection methods (MSS, DGC, and Swim-up). The authors found that ORP was significantly lower in the microfluidic group compared to the other groups. However, a comparison with our results is not possible, as our method measures the occurrence of intracytoplasmic sperm oxidation, whereas ORP measures the balance between total oxidants and reductants in semen.

To our knowledge, this is the first study to apply microfluidic selection to semen samples with hyperviscosity, demonstrating that it is an effective strategy for handling samples with this anomaly. Indeed, it has been reported that ICSI outcomes are poorer in infertile couples with increased seminal viscosity compared to those with normal viscosity [74], likely because DGC or Swim-up techniques do not always ensure the recovery of a sufficient number or quality of highly motile spermatozoa free from contamination by immotile spermatozoa or other cell types.

To date, only a few studies have evaluated the effect of microfluidic selection devices in non-normozoospermic semen samples. All studies support that MSS yields better results in terms of sperm motility, viability, and low sDF compared to DGC and Swim-up [32,33,34], consistent with the findings of the present study. In particular, Vahidi et al. [33] demonstrated that MSS is more effective in selecting high-quality spermatozoa than other non-conventional methods, such as zeta potential or MACS. However, these studies [32,33,34] concluded that MSS is not suitable for cases of oligozoospermia due to very low yields. In our case, similar sperm concentrations, starting from the same semen volume, were recovered after both Swim-up and MSS. A case report on a couple with secondary infertility due to oligoasthenozoospermia in the male partner showed that sperm preparation with a microfluidic device resulted in a clinical pregnancy after ICSI [75]. Overall, these studies suggest that MSS selection could be particularly beneficial in cases of male factor infertility. It should be noted that although improvements in sperm parameters such as motility may appear modest (although statistically significant), they can be biologically important, particularly in the context of ART. The ability to select spermatozoa with optimal functional quality becomes especially relevant when working with compromised semen samples—such as those with poor quality (e.g., oligozoospermia, teratozoospermia, or asthenozoospermia), high viscosity, or elevated DNA fragmentation. In these cases, standard selection methods commonly used in fertility clinics (Swim-up or DGC) may not effectively isolate the most competent spermatozoa, likely because viscosity does not allow correct sperm swimming and/or because sperm DNA is particularly vulnerable to damage during such procedures. Therefore, even slight improvements in sperm parameters may reflect the enrichment of a higher-quality sperm subpopulation, with potentially beneficial effects on fertilization outcomes and embryo development.

A potential limitation of this study is that although we followed the recommended incubation times for each method, these differ between MSS (30 min) and SU (60 min). A longer incubation time could, on one hand, improve the yield in terms of sperm count, but, on the other hand, it could also select spermatozoa with suboptimal semen quality. However, our findings suggest that extending the incubation time of SU does not negatively impact sperm quality, as no significant differences were observed in sperm motility, viability, or DNA fragmentation between the two durations (Figure 8).

Another limitation of the study is that only a subset of samples was analyzed for sperm oxidative levels and DNA fragmentation due to the limited number of spermatozoa recovered after the selection process.

Several studies provided data on the application of microfluidics in ART procedures; however, as mentioned above, whether ART outcomes improve with MSS sperm selection is still unclear. A recent meta-analysis reported a slight enhancement in ART outcomes, including fertilization, cleavage, blastocyst rate, and clinical pregnancy, as well as a reduction in miscarriage rates, although without statistical significance [35]. Studies by Palermo and co-authors [36,37,38] support the use of this selection method to increase the number of euploid embryos and improve clinical pregnancy rates in couples who experienced previous ICSI failure due to high embryo aneuploidy rates. These findings emphasize the importance of careful patient selection before applying MSS in ART procedures. Beyond couples with prior adverse outcomes, our study suggests that MSS may be beneficial for cases of asthenozoospermia, oligozoospermia, teratozoospermia, and semen hyperviscosity. Additionally, as previously mentioned, MSS selection could also be advantageous in first-line ART techniques.

## 5. Conclusions

Our study demonstrated that the microfluidic sorting is an effective method for selecting the optimal sperm fraction, not only in normozoospermic samples but also in semen samples exhibiting defects, such as hyperviscosity, a low concentration, reduced motility, abnormal morphology, and high sDF levels. Moreover, this selection procedure is significantly faster than traditional methods, preparing spermatozoa for use in half the time. Additionally, the potential for operator-induced variability is greatly reduced, as the sample undergoes much less manipulation. However, the technology is currently quite expensive, making it more suitable for samples where traditional techniques fail to yield satisfactory results. Overall, the application of microfluidics could improve chances of successful fertilization and lead to better reproductive outcomes, especially for individuals with compromised semen quality. Future research should focus on prospective clinical trials aimed at evaluating the impact of MSS on ART outcomes. Additionally, larger studies involving patients with different types of semen abnormalities will be essential to better define the specific patient populations that could benefit most from MSS. These studies will contribute to a more comprehensive understanding of the clinical value of MSS and its potential role in improving ART success rates.

## Figures and Tables

**Table 1:** Median and [IQR] values of age, sexual abstinence, and semen parameters of all normozoospermic samples included in the study (*n* = 57), and after dividing into two groups based on viscosity (normal viscosity, *n* = 26, and hyperviscosity, *n* = 31). *p* = not significant (ns), non-viscous vs. hyper-viscous semen samples.

	Age (Years)	Abstinence (Days)	Volume (mL)	pH	Progressive Motility (%)	Total Motility (%)	Concentration (×10^6^/mL)	Normal Morphology (%)
All samples (*n* = 57)	34 [28–37]	4 [3–6]	5.1 [4.1–5.6]	7.8 [7.6–7.8]	62.0 [56.0–67.5]	66.0 [63.0–71.0]	77.0 [52.0–119.5]	5.0 [4.0–7.0]
Normal viscosity (*n* = 26)	35.0 [30.5–37.0]	5.0 [3.7–6.0]	5.2 [4.5–5.7]	7.8 [7.6–7.8]	63.0 [57.5–67.5]	66.5 [64.0–73.3]	87.8 [59.8–172.5]	5.0 [4.0–8.0]
Hyperviscosity (*n* = 31)	33.0 [24.0–36.0]	4.0 [3.0–6.0]	4.9 [4.0–5.6]	7.8 [7.6–8.0]	61.0 [55.0–68.0]	66.0 [60.0–71.0]	71.0 [48.0–116.0]	5.0 [4.0–6.0]
*p*	ns	ns	ns	ns	ns	ns	ns	ns

**Table 2:** Median and [IQR] values of kinematic sperm parameters, including VAP, VSL, VCL, ALH, BCF, LIN, and STR in Unselected, SU, and MSS selected samples, for the normal viscosity (*n* = 26) and hyperviscosity (*n* = 31) groups. * *p* < 0.001 vs. Unselected; § *p* < 0.05 vs. Unselected; ^ *p* < 0.001 vs. SU selected; ° *p* < 0.05 vs. SU selected.

		*VAP (µm/s)*	*VSL (µm/s)*	*VCL (µm/s)*	*ALH (µm)*	*BCF (Hz)*	*LIN (%)*	*STR (%)*
***Normal viscosity***	***Unselected***	37.2 [33.9–40.6]	28.2 [24.0–31.1]	57.9 [35.1–66.4]	4.2 [3.7–4.6]	21.5 [20.3–24.6]	45.0 [41.4–48.3]	72.1 [68.0–75.5]
	***SU selected***	62.6 [55.7–72.2] *	49.1 [41.3–57.3] *	100.9 [92.4–126.1] *	5.8 [5.1–6.9] *	23.0 [21.0–24.8]	49.3 [38.7–53.8]	77.6 [70.8–80.1] §
	***MSS selected***	67.8 [55.5–73.0] *°	49.0 [44.9–62.2] *°	108.9 [92.1–128.4] *	6.2 [5.4–7.6]*	21.1 [19.2–22.6] ^	50.8 [44.1–55.1] §	77.4 [72.9–81.0] *
***Hyperviscosity***	***Unselected***	40.8 [31.0–44.0]	31.0 [22.0–35.1]	61.4 [51.8–70.5]	3.9 [3.5–4.6]	24.2 [21.5–26.4]	44.6 [39.2–50.1]	70.7 [66.9–77.7]
	***SU selected***	62.2 [48.2–73.4] *	45.3 [37.3–55.5] *	108.2 [82.2–141.7] *	5.8 [4.2–8.0] *	24.8 [22.2–28.6]	44.1 [40.5–51.8]	74.4 [68.9–80.3] §
	***MSS selected***	68.2 [62.4–76.9] *°	56.1 [47.3–62.9] *^	113.2 [105.1–136.2] *°	6.8 [5.6–7.7] *°	22.3 [20.1–23.5] §°	46.9 [42.9–53.6] §°	77.5 [73.3–82.7] *°

**Table 3:** Median and [IQR] values of sperm progressive motility (*n* = 18), total motility (*n* = 18), viability (*n* = 18), CellROX^®^ Orange sperm positivity (*n* = 9), and PI brighter, PI dimmer, and total sDF (*n* = 14) in Unselected, Direct SU, SU, and MSS selected samples. § *p* < 0.05 vs. Unselected; * *p* < 0.001 vs. Unselected; ° *p* < 0.05 vs. SU selected; ^ *p* < 0.001 vs. SU selected; # *p* < 0.05 vs. Direct SU selected; @ *p* < 0.001 vs. Direct SU selected.

	Progressive Motility (%)	Total Motility (%)	Viability (%)	CellROX^®^ Orange Positivity (%)	sDF (%)		
					Pi Brighter	Pi Dimmer	Total
**Unselected**	63.5 [55.5–69.5]	67.5 [60.8–74.5]	83.0 [73.8–91.3]	9.8 [8.4–20.5]	24.2 [19.6–28.7]	11.2 [7.7–12.8]	34.4 [30.0–38.7]
**Direct SU** **selected**	85.5 [82.8–90.3] *	90.0 [86.0–94.0] *	96.5 [92.8–98.0] §	11.4 [8.4–25.3]	12.2 [8.3–15.2] *	1.1 [0.4–3.5] *	12.8 [9.1–17.3] *
**SU selected**	85.5 [76.8–91.0] *	89.0 [78.0–94.3] *#	94.0 [87.3–96.5] *#	17.8 [4.8–30.7]	20.9 [13.2–24.6] §#	5.4 [1.2–8.5] §#	29.0 [18.2–33.3] §#
**MSS selected**	93.0 [90.0–95.0] *°@	96.0 [93.8–97.3] *^@	100.0 [99.8–100.0] *^@	22.7 [9.1–29.6]	9.9 [5.6–12.8] *°	0.3 [0.2–0.6] *#°	10.3 [6.0–14.1] *#^

**Table 4:** Median and [IQR] values of age, sexual abstinence, and semen parameters of 15 oligozoospermic and 9 asthenozoospermic samples included in the study.

	Age (Years)	Abstinence (Days)	Volume (mL)	pH	Progressive Motility (%)	Total Motility (%)	Concentration (×10^6^/mL)	Normal Morphology (%)
**Oligozoospermic samples (*n* = 15)**	33.0 [26.0–37.0]	3.0 [3.0–5.0]	4.5 [3.8–5.8]	7.8 [7.6–7.8]	40.0 [33.0–57.0]	50.0 [43.0–64.0]	6.2 [4.1–8.8]	2.0 [1.0–4.0]
**Asthenozoospermic samples (*n* = 9)**	40.0 [28.5–44.0]	4.0 [3.0–5.0]	5.0 [4.3–6.1]	7.8 [7.6–7.8]	15.0 [11.5–30.5]	30.0 [18.0–41.5]	15.5 [8.1–19.0]	1.0 [0.0–2.0]

## Data Availability

The data that support the findings of this study are available on request from the corresponding author. The data are not publicly available due to privacy or ethical restrictions.

## References

[1] Pinto S., Carrageta D.F., Alves M.G., Rocha A., Agarwal A., Barros A., Oliveira P.F. (2021). Sperm selection strategies and their impact on assisted reproductive technology outcomes. Andrologia.

[2] Leung E.T.Y., Lee C.L., Tian X., Lam K.K.W., Li R.H.W., Ng E.H.Y., Yeung W.S.B., Chiu P.C.N. (2022). Simulating nature in sperm selection for assisted reproduction. Nat. Rev. Urol..

[3] Doostabadi M.R., Mangoli E., Marvast L.D., Dehghanpour F., Maleki B., Torkashvand H., Talebi A.R. (2022). Microfluidic devices employing chemo- and thermotaxis for sperm selection can improve sperm parameters and function in patients with high DNA fragmentation. Andrologia.

[4] Akerlöf E., Fredricson B., Gustafsson O., Lundin A., Lunell N.O., Nylund L., Rosenborg L., Pousette A. (1987). Comparison between a swim-up and a Percoll gradient technique for the separation of human spermatozoa. Int. J. Androl..

[5] WHO (2021). WHO Laboratory Manual for the Examination and Processing of Human Semen.

[6] Agarwal A., Ikemoto I., Loughlin K.R. (1994). Effect of sperm washing on levels of reactive oxygen species in semen. Arch. Androl..

[7] Rappa K.L., Rodriguez H.F., Hakkarainen G.C., Anchan R.M., Mutter G.L., Asghar W. (2016). Sperm processing for advanced reproductive technologies: Where are we today?. Biotechnol. Adv..

[8] Zini A., Finelli A., Phang D., Jarvi K. (2000). Influence of semen processing technique on human sperm DNA integrity. Urology.

[9] Muratori M., Tarozzi N., Cambi M., Boni L., Iorio A.L., Passaro C., Luppino B., Nadalini M., Marchiani S., Tamburrino L. (2016). Variation of DNA Fragmentation Levels During Density Gradient Sperm Selection for Assisted Reproduction Techniques: A Possible New Male Predictive Parameter of Pregnancy?. Medicine.

[10] Muratori M., Tarozzi N., Carpentiero F., Danti S., Perrone F.M., Cambi M., Casini A., Azzari C., Boni L., Maggi M. (2019). Sperm selection with density gradient centrifugation and swim up: Effect on DNA fragmentation in viable spermatozoa. Sci. Rep..

[11] Kim S.W., Jee B.C., Kim S.K., Kim S.H. (2017). Sperm DNA fragmentation and sex chromosome aneuploidy after swim-up versus density gradient centrifugation. Clin. Exp. Reprod. Med..

[12] Aitken R.J., Clarkson J.S. (1988). Significance of reactive oxygen species and antioxidants in defining the efficacy of sperm preparation techniques. J. Androl..

[13] Katigbak R.D., Turchini G.M., de Graaf S.P., Kong L., Dumée L.F. (2019). Review on Sperm Sorting Technologies and Sperm Properties toward New Separation Methods via the Interface of Biochemistry and Material Science. Adv. Biosyst..

[14] Ahmadkhani N., Hosseini M., Saadatmand M., Abbaspourrad A. (2022). The influence of the female reproductive tract and sperm features on the design of microfluidic sperm-sorting devices. J. Assist. Reprod. Genet..

[15] Schuster T.G., Cho B., Keller L.M., Takayama S., Smith G.D. (2003). Isolation of motile spermatozoa from semen samples using microfluidics. Reprod. Biomed. Online.

[16] Cho B.S., Schuster T.G., Zhu X., Chang D., Smith G.D., Takayama S. (2003). Passively driven integrated microfluidic system for separation of motile sperm. Anal. Chem..

[17] Shirota K., Yotsumoto F., Itoh H., Obama H., Hidaka N., Nakajima K., Miyamoto S. (2016). Separation efficiency of a microfluidic sperm sorter to minimize sperm DNA damage. Fertil. Steril..

[18] Sarbandi I.R., Lesani A., Moghimi Zand M., Nosrati R. (2021). Rheotaxis-based sperm separation using a biomimicry microfluidic device. Sci. Rep..

[19] Sharma S., Kabir M.A., Asghar W. (2022). Selection of healthy sperm based on positive rheotaxis using a microfluidic device. Analyst.

[20] Zaferani M., Cheong S.H., Abbaspourrad A. (2018). Rheotaxis-based separation of sperm with progressive motility using a microfluidic corral system. Proc. Natl. Acad. Sci. USA.

[21] Huang C.H., Chen C.H., Huang T.K., Lu F., Jen Huang J.Y., Li B.R. (2023). Design of a gradient-rheotaxis microfluidic chip for sorting of high-quality Sperm with progressive motility. iScience.

[22] Koyama S., Amarie D., Soini H.A., Novotny M.V., Jacobson S.C. (2006). Chemotaxis assays of mouse sperm on microfluidic devices. Anal. Chem..

[23] Xie L., Ma R., Han C., Su K., Zhang Q., Qiu T., Wang L., Huang G., Qiao J., Wang J. (2010). Integration of sperm motility and chemotaxis screening with a microchannel-based device. Clin. Chem..

[24] Zhang Y., Xiao R.R., Yin T., Zou W., Tang Y., Ding J., Yang J. (2015). Generation of Gradients on a Microfluidic Device: Toward a High-Throughput Investigation of Spermatozoa Chemotaxis. PLoS ONE.

[25] Li K., Li R., Ni Y., Sun P., Liu Y., Zhang D., Huang H. (2018). Novel distance-progesterone-combined selection approach improves human sperm quality. J. Transl. Med..

[26] Li Z., Liu W., Qiu T., Xie L., Chen W., Liu R., Lu Y., Mitchelson K., Wang J., Qiao J. (2014). The construction of an interfacial valve-based microfluidic chip for thermotaxis evaluation of human sperm. Biomicrofluidics.

[27] Pérez-Cerezales S., Laguna-Barraza R., de Castro A.C., Sánchez-Calabuig M.J., Cano-Oliva E., de Castro-Pita F.J., Montoro-Buils L., Pericuesta E., Fernández-González R., Gutiérrez-Adán A. (2018). Sperm selection by thermotaxis improves ICSI outcome in mice. Sci. Rep..

[28] Ko Y.J., Maeng J.H., Hwang S.Y., Ahn Y. (2018). Design, Fabrication, and Testing of a Microfluidic Device for Thermotaxis and Chemotaxis Assays of Sperm. SLAS Technol..

[29] Yan Y., Zhang B., Fu Q., Wu J., Liu R. (2021). A fully integrated biomimetic microfluidic device for evaluation of sperm response to thermotaxis and chemotaxis. Lab Chip.

[30] Gode F., Gürbüz A.S., Tamer B., Pala I., Isik A.Z. (2020). The Effects of Microfluidic Sperm Sorting, Density Gradient and Swim-up Methods on Semen Oxidation Reduction Potential. Urol. J..

[31] Pujol A., García-Peiró A., Ribas-Maynou J., Lafuente R., Mataró D., Vassena R. (2022). A microfluidic sperm-sorting device reduces the proportion of sperm with double-stranded DNA fragmentation. Zygote.

[32] Hsu C.T., Lee C.I., Lin F.S., Wang F.Z., Chang H.C., Wang T.E., Huang C.C., Tsao H.M., Lee M.S., Agarwal A. (2023). Live motile sperm sorting device for enhanced sperm-fertilization competency: Comparative analysis with density-gradient centrifugation and microfluidic sperm sorting. J. Assist. Reprod. Genet..

[33] Vahidi N., Eyni H., Sabz F.T.K., Narimani N., Zandieh Z., Amjadi F. (2024). Microfluidic in compared with Zeta potential, MACS and swim up methods, resulted in improved chromatin integrity and high quality sperms. JBRA Assist. Reprod..

[34] Sheibak N., Amjadi F., Shamloo A., Zarei F., Zandieh Z. (2024). Microfluidic sperm sorting selects a subpopulation of high-quality sperm with a higher potential for fertilization. Hum. Reprod..

[35] Ferreira Aderaldo J., da Silva Maranhão K., Ferreira Lanza D.C. (2023). Does microfluidic sperm selection improve clinical pregnancy and miscarriage outcomes in assisted reproductive treatments? A systematic review and meta-analysis. PLoS ONE.

[36] Parrella A., Keating D., Cheung S., Xie P., Stewart J.D., Rosenwaks Z., Palermo G.D. (2019). A treatment approach for couples with disrupted sperm DNA integrity and recurrent ART failure. J. Assist. Reprod. Genet..

[37] Kocur O.M., Xie P., Souness S., Cheung S., Rosenwaks Z., Palermo G.D. (2023). Assessing male gamete genome integrity to ameliorate poor assisted reproductive technology clinical outcome. F S Sci..

[38] Kocur O.M., Xie P., Cheung S., Souness S., McKnight M., Rosenwaks Z., Palermo G.D. (2023). Can a sperm selection technique improve embryo ploidy?. Andrology.

[39] Banti M., Van Zyl E., Kafetzis D. (2024). Sperm Preparation with Microfluidic Sperm Sorting Chip May Improve Intracytoplasmic Sperm Injection Outcomes Compared to Density Gradient Centrifugation. Reprod. Sci..

[40] Traini G., Tamburrino L., Vignozzi L., Baldi E., Marchiani S. (2022). Is oxidative stress evaluated in viable human spermatozoa a marker of good semen quality?. Front. Endocrinol..

[41] Muratori M., Marchiani S., Tamburrino L., Tocci V., Failli P., Forti G., Baldi E. (2008). Nuclear staining identifies two populations of human sperm with different DNA fragmentation extent and relationship with semen parameters. Hum. Reprod..

[42] Mortimer S.T., Swan M.A., Mortimer D. (1998). Effect of seminal plasma on capacitation and hyperactivation in human spermatozoa. Hum. Reprod..

[43] Kazerooni T., Asadi N., Jadid L., Kazerooni M., Ghanadi A., Ghaffarpasand F., Kazerooni Y., Zolghadr J. (2009). Evaluation of sperm’s chromatin quality with acridine orange test, chromomycin A3 and aniline blue staining in couples with unexplained recurrent abortion. J. Assist. Reprod. Genet..

[44] Marchiani S., Tamburrino L., Olivito B., Betti L., Azzari C., Forti G., Baldi E., Muratori M. (2014). Characterization and sorting of flow cytometric populations in human semen. Andrology.

[45] Muratori M., Marchiani S., Tamburrino L., Cambi M., Lotti F., Natali I., Filimberti E., Noci I., Forti G., Maggi M. (2015). DNA fragmentation in brighter sperm predicts male fertility independently from age and semen parameters. Fertil. Steril..

[46] Ogle R.A., Netherton J., Schneider E., Velkov T., Zhang H., Cole N., Hetherington L., Villaverde A.I.S.B., Baker M.A. (2021). Nuclear heterogeneity is prevalent in high-quality fractionated human sperm cells typically used for assisted conception. Hum. Reprod..

[47] René C., Landry I., de Montigny F. (2021). Couples’ experiences of pregnancy resulting from assisted reproductive technologies: A qualitative meta-synthesis. Int. J. Nurs. Stud. Adv..

[48] Anbari F., Khalili M.A., Sultan Ahamed A.M., Mangoli E., Nabi A., Dehghanpour F., Sabour M. (2021). Microfluidic sperm selection yields higher sperm quality compared to conventional method in ICSI program: A pilot study. Syst. Biol. Reprod. Med..

[49] Feyzioglu B.S., Avul Z. (2023). Effects of sperm separation methods before intrauterine insemination on pregnancy outcomes and live birth rates: Differences between the swim-up and microfluidic chip techniques. Medicine.

[50] Zaha I., Naghi P., Stefan L., Bunescu C., Radu M., Muresan M.E., Sandor M., Sachelarie L., Huniadi A. (2023). Comparative Study of Sperm Selection Techniques for Pregnancy Rates in an Unselected IVF-ICSI Population. J. Pers. Med..

[51] Quinn M.M., Jalalian L., Ribeiro S., Ona K., Demirci U., Cedars M.I., Rosen M.P. (2018). Microfluidic sorting selects sperm for clinical use with reduced DNA damage compared with density gradient centrifugation with swim-up in split semen samples. Hum. Reprod..

[52] Aydın Ş., Bulgan Kılıçdağ E., Çağlar Aytaç P., Çok T., Şimşek E., Haydardedeoğlu B. (2022). Prospective randomized controlled study of a microfluidic chip technology for sperm selection in male infertility patients. Andrologia.

[53] Delaroche L., Caillou H., Lamazou F., Genauzeau E., Meicler P., Oger P., Dupont C., Humaidan P. (2020). Live birth after intrauterine insemination: Is there an upper cut-off for the number of motile spermatozoa inseminated?. Reprod. Biomed. Online.

[54] Zhao J., Zhang Q., Wang Y., Li Y. (2014). Whether sperm deoxyribonucleic acid fragmentation has an effect on pregnancy and miscarriage after in vitro fertilization/intracytoplasmic sperm injection: A systematic review and meta-analysis. Fertil. Steril..

[55] Cissen M., Wely M.V., Scholten I., Mansell S., Bruin J.P., Mol B.W., Braat D., Repping S., Hamer G. (2016). Measuring Sperm DNA Fragmentation and Clinical Outcomes of Medically Assisted Reproduction: A Systematic Review and Meta-Analysis. PLoS ONE.

[56] Robinson L., Gallos I.D., Conner S.J., Rajkhowa M., Miller D., Lewis S., Kirkman-Brown J., Coomarasamy A. (2012). The effect of sperm DNA fragmentation on miscarriage rates: A systematic review and meta-analysis. Hum. Reprod..

[57] Tamburrino L., Traini G., Ragosta M.E., Dabizzi S., Vezzani S., Scarpa F., Vignozzi L., Baldi E., Marchiani S. (2024). Semen cryopreservation and storage in liquid nitrogen: Impact on chromatin compaction. Andrology.

[58] Cho C.L., Agarwal A. (2017). Role of sperm DNA fragmentation in male factor infertility: A systematic review. Arab. J. Urol..

[59] Alahmar A.T., Singh R., Palani A. (2022). Sperm DNA Fragmentation in Reproductive Medicine: A Review. J. Hum. Reprod. Sci..

[60] Agarwal A., Said T.M. (2003). Role of sperm chromatin abnormalities and DNA damage in male infertility. Hum. Reprod. Update.

[61] Esteves S.C., Agarwal A., Majzoub A. (2017). An evidence-based perspective on the role of sperm chromatin integrity and sperm DNA fragmentation testing in male infertility. Transl. Androl. Urol..

[62] Le M.T., Dang H.N.T., Nguyen T.V., Nguyen T.T.T., Nguyen Q.H.V., Cao N.T. (2022). Effects of sperm preparation techniques on sperm survivability and DNA fragmentation. J. Int. Med. Res..

[63] Amano K., Oigawa S., Ichizawa K., Tokuda Y., Unagami M., Sekiguchi M., Furui M., Nakaoka K., Ito A., Hayashi R. (2024). Swim-up method is superior to density gradient centrifugation for preserving sperm DNA integrity during sperm processing. Reprod. Med. Biol..

[64] Wang M., Sun J., Wang L., Gao X., Lu X., Wu Z., Wang Y., Liu K., Tao J., Wu Y. (2014). Assessment of density gradient centrifugation (DGC) and sperm chromatin dispersion (SCD) measurements in couples with male factor infertility undergoing ICSI. J. Assist. Reprod. Genet..

[65] Aitken R.J., Finnie J.M., Muscio L., Whiting S., Connaughton H.S., Kuczera L., Rothkirch T.B., De Iuliis G.N. (2014). Potential importance of transition metals in the induction of DNA damage by sperm preparation media. Hum. Reprod..

[66] Mirsanei J.S., Sheibak N., Zandieh Z., Mehdizadeh M., Aflatoonian R., Tabatabaei M., Mousavi A.S., Amjadi F. (2022). Microfluidic chips as a method for sperm selection improve fertilization rate in couples with fertilization failure. Arch. Gynecol. Obstet..

[67] Odhiambo J.F., DeJarnette J.M., Geary T.W., Kennedy C.E., Suarez S.S., Sutovsky M., Sutovsky P. (2014). Increased conception rates in beef cattle inseminated with nanopurified bull semen. Biol. Reprod..

[68] Omar M.A., Raouf K.S., ElGuindy T., Kamel N., Arafa S.S., Gebril A. (2020). Magnetic-activated sperm enrichment (MASE) versus density gradient centrifugation (DGC) impact on ICSI outcome. IJGO.

[69] Agarwal A., Virk G., Ong C., du Plessis S.S. (2014). Effect of oxidative stress on male reproduction. World J. Men’s Health.

[70] Agarwal A., Sharma R.K., Nallella K.P., Thomas A.J., Alvarez J.G., Sikka S.C. (2006). Reactive oxygen species as an independent marker of male factor infertility. Fertil. Steril..

[71] de Lamirande E., Jiang H., Zini A., Kodama H., Gagnon C. (1997). Reactive oxygen species and sperm physiology. Rev. Reprod..

[72] Chen S.J., Allam J.P., Duan Y.G., Haidl G. (2013). Influence of reactive oxygen species on human sperm functions and fertilizing capacity including therapeutical approaches. Arch. Gynecol. Obstet..

[73] Wang Y., Fu X., Li H. (2025). Mechanisms of oxidative stress-induced sperm dysfunction. Front. Endocrinol..

[74] Esfandiari N., Burjaq H., Gotlieb L., Casper R.F. (2008). Seminal hyperviscosity is associated with poor outcome of in vitro fertilization and embryo transfer: A prospective study. Fertil. Steril..

[75] Bedwal R.G., Nair N., Pareek C., More A., Kalbande A. (2024). Enhancing the Fertility Potential: A Case Report on the Management of Oligoasthenozoospermia Using a Microfluidic Device. Cureus.

